# Impact of Front-of-Pack Nutrition Labels on Portion Size Selection: An Experimental Study in a French Cohort

**DOI:** 10.3390/nu10091268

**Published:** 2018-09-08

**Authors:** Manon Egnell, Emmanuelle Kesse-Guyot, Pilar Galan, Mathilde Touvier, Mike Rayner, Jo Jewell, João Breda, Serge Hercberg, Chantal Julia

**Affiliations:** 1 Nutritional Epidemiology Research Team (EREN), Sorbonne Paris Cité Epidemiology and Statistics Research Center (CRESS), U1153 Inserm, U1125, Inra, Cnam, Paris 13 University, 93000 Bobigny, France; e.kesse@eren.smbh.univ-paris13.fr (E.K.-G.); p.galan@eren.smbh.univ-paris13.fr (P.G.); m.touvier@eren.smbh.univ-paris13.fr (M.T.); s.hercberg@uren.smbh.univ-paris13.fr (S.H.); c.julia@uren.smbh.univ-paris13.fr (C.J.); 2 Centre on Population Approaches for Non-Communicable Disease Prevention, University of Oxford, Oxford OX1 2JD, UK; mike.rayner@dph.ox.ac.uk; 3 Nutrition, Physical Activity and Obesity Programme, WHO Regional Office for Europe, DK-2100 Copenhagen, Denmark; jewellj@who.int (J.J.); rodriguesdasilvabred@who.int (J.B.); 4 Office for the Prevention and Control of Noncommunicable Diseases, WHO Regional Office for Europe, 125009 Moscow, Russia; 5 Public Health Department, Avicenne Hospital, Assistance Publique des Hôpitaux de Paris (AP-HP), 93000 Bobigny, France

**Keywords:** Front-of-Pack nutrition label, portion size, food products

## Abstract

In the European Union (EU) three coloured graded Front-of-Pack labels (FoPLs), two endorsed by governments (Nutri-Score and Multiple Traffic Lights (MTL)) and one designed by industry (Evolved Nutrition Label (ENL)) are currently being discussed. This study aimed to investigate the impact of these FoPLs on portion size selection, specifically for less healthy products. In 2018, participants from the French NutriNet-Santé cohort study (*N* = 25,772) were exposed through a web-based self-administered questionnaire to products from three food categories (sweet biscuits, cheeses, and sweet spreads), with or without FoPLs, and were invited to select the portion they would consume (in size and number). Kruskall-Wallis tests, and mixed ordinal logistic regression models, were used to investigate the effects of FoPLs on portion size selection. Compared to no label, Nutri-Score consistently lowered portion sizes (OR = 0.76 (0.74–0.76)), followed by MTL (OR = 0.83 (0.82–0.84)). For ENL, the effects differed depending on the food group: It lowered portion size selection for cheeses (OR = 0.84 (0.83–0.87)), and increased it for spreads (OR = 1.19 (1.15–1.22)). Nutri-Score followed by MTL appear efficient tools to encourage consumers to decrease their portion size for less healthy products, while ENL appears to have inconsistent effects depending on the food category.

## 1. Introduction

Various Front-of-Pack nutrition labels (FoPLs), varying both in the graphical design and the information they convey, have been implemented in Europe and internationally, in order to provide information on the nutritional quality of food products and to help consumers identify healthier foods [1]. Given the voluntary nature of the measure in multiple countries, and in particular in the European Union (EU), multiple schemes currently co-exist within a single market, which could lead to confusion for consumers [2]. Within the EU market, several voluntary FoPLs have been developed and/or endorsed by governments with a view to encourage their uptake by manufacturers and retailers. FoPLs formats can be divided into nutrient-specific labels, such as the Multiple Traffic Lights (MTL) implemented in the United Kingdom since 2004 [3], and summary systems, such as the Green Keyhole used in Sweden since 1980, and then in Nordic countries [4], or the Nutri-Score adopted in October 2017 in France [5,6]. The food industry has long opposed interpretative FoPLs (i.e., that use graphics, symbols or colours), though they have been demonstrated to be better understood by consumers than non-interpretative labels with numeric information only, such as the industry-led Reference Intakes scheme [7].

However, a consortium of six agro-industry firms recently proposed an interpretive FoP nutrition labelling, the Evolved Nutrition Label (ENL), which is an adaptation of the Multiple Traffic Lights system [8]. MTL are a nutrient-specific FoP nutrition label, providing numeric information for fats, saturated fatty acids, sugars and salt and an assessment of these amounts based on semantic colours: Red for high amounts, amber for medium, and green for low amounts. The adaptation from MTL to ENL relies on the modification of the settings for the amber/red threshold, specifically for products with small portion size (set at ≤60 g), from a 100 g basis to a per portion basis. In practice, the implication is that food products with high amounts of unfavourable nutrients per 100 g (fats, saturated fatty acids, sugars, and salt) may see their FoPL switch from red to amber, compared with MTL, if the portion size is sufficiently low. Products that are mostly impacted by this modification are generally foods with lower nutritional quality and consumed theoretically in small portion size, for which ENL tends to provide more favourable assessments than MTL (red in MTL and amber in ENL). One of the arguments put forward by the manufacturers is that the ENL would encourage the industry to reformulate and provide smaller portion sizes, and also give to the consumers more accurate information regarding the composition of the actual portion they consume. However, no evidence has been provided to support this hypothesis, and opinions are much contrasted. Indeed, this alternative has notably been criticised by European consumer organisations, who believe the ENL would make it harder for consumers to compare different products’ healthiness, and misleads them on the nutritional quality of foods [9].

In France, the Nutri-Score, a summary colour-coded and graded FoP nutrition label, adopted in 2017 by health authorities to be applied on pre-packed food products, indicates the overall nutritional quality of a food product based on 100g [5,6]. Multiple studies have validated the positive effect of the Nutri-Score on various dimensions, including perception, objective understanding, and nutritional quality of food purchases [10,11,12,13,14].

Very few studies have investigated the impact of FoP nutritional labelling on portion size selection [15,16,17], while a review has suggested that they may modify the perception of consumers of the nutritional quality of products, and then lead to “halo” effect, influencing the quantities consumed [18]. Indeed, misinterpretations of the actual nutritional content of a food may lead consumers towards larger portions and overconsumption of less healthy foods [19]. Therefore, it appears of major importance to study the effects of FoP nutrition labels on portion size selection, and in particular for less healthy options, for which a reduction in the portion size would be of interest.

The objective of the present study was to assess comparatively the effect of the Evolved Nutrition Label, Multiple Traffic Lights and Nutri-Score, on the portion size selection of less healthy food products, compared to a control situation without any FoP nutrition label.

## 2. Methods

### 2.1. Participants

Participants were recruited from the French NutriNet-Santé cohort, an ongoing web-based cohort of adult volunteers, launched in France in 2009 [20]. At baseline and during the follow-up, participants of the cohort are invited to complete a set of questionnaires to collect data on sociodemographic and lifestyle characteristics, dietary intakes, health status, anthropometric measurements, and physical activity. During follow-up, volunteers are also invited to complete additional and optional questionnaires, pertaining to various aspects of dietary behaviours and health determinants. The NutriNet-Santé study is conducted according to the French Institute for Health and Medical Research (IRB Inserm no. 0000388FWA00005831) and the “Commission Nationale de l’Informatique et des Libertés” (CNIL n°908450/n°909216). Electronic informed consent was obtained from each participant. For the present study, a specific questionnaire was developed and sent to the participants of the cohort in April 2018. The questionnaire was optional for participants and available for a limited period of time. For this specific study, we aimed at including a target number of *N* = 25,000 participants from the overall study, in order to achieve a balance between statistical power, with an expected small effect size, and the relevance of observed differences. Participants did not receive any form of incentive or compensation to participate in the online survey. Participants who opened the questionnaire but did not complete and validate it were considered as excluded participants.

### 2.2. Stimuli

#### 2.2.1. Front-of-Pack Labels Tested

**Figure 1:**
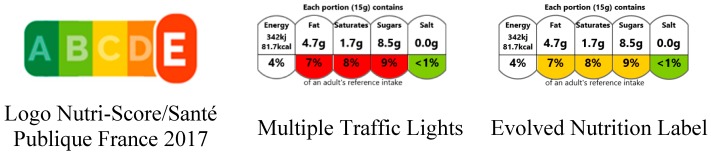
Example of the three Front-of-Pack labels (FoPLs) for a single food product tested in the study (a chocolate spread).

Three different FoPLs were tested, including the Evolved Nutrition Label, the Multiple Traffic Lights and the Nutri-Score (Figure 1). In addition, a no label condition was used in order to compare all FoPLs with a control situation. The labels were presented on actual food packages, with an additional presentation below the food, to ensure a high visibility.

The Nutri-Score is a summary, graded, colour-coded FoPL adopted by health authorities in France in October 2017 to be applied on pre-packaged food products of the French market [6]. This voluntary FoPL, providing information on the overall nutritional quality of a food product using a 5-scale scheme, is based on the United Kingdom (UK) Food Standard Agency Nutrient Profiling System, modified by the French Health Council of Public Health (FSAm-NPS) [21]. The FSAm-NPS score, ranging from −15 to +40 points, results from the difference between positive points attributed to unfavourable nutrients (i.e., energy (kJ), saturated fatty acids (g), total sugars (g) and sodium (mg), 0 to 10 points for each), and negative points for favourable nutrients (i.e., protein (g), fibre (g) and percentage of fruits, vegetables and nuts (%), 0 to 5 points for each), for 100 g of food product. Lower FSAm-NPS reflects higher nutritional quality of food or beverage. The FSAm-NPS of a food or beverage is then translated into the Nutri-Score scale, from A/Green to E/Red.

The Multiple Traffic Lights is a nutrient-specific FoPL, implemented by the Food Standard Agency in UK since 2004 [3], and providing numerical information on the amounts of energy, lipids, saturated fat, sugars and salt for a portion of food products, as well as their contribution to an adult’s daily reference intakes. In addition, an assessment of the amounts of nutrients is provided through the attribution of a colour for the nutrient content, except energy: Red for high content, amber for medium and green for low content. The colour allocation is based on the amount of nutrients per 100 g, except for the ‘amber’/’red’ threshold, which can use a per portion basis for foods with a portion over 100 g or 150 mL only.

The Evolved Nutrition Label, recently proposed by a consortium of five agro-industry firms [8], is a modified version of the MTL. The ENL indicates also the amounts of energy, lipids, sugars and salt per portion of food products, with associated colours. However, for products with small portions, in contrast to the MTL, the attribution of colours between amber and red is based on the nutrient content for a portion of product (defined by an industry-led taskforce) and not for 100 g. More specifically, for products in small portions (set at ≤60 g), the threshold for the red/amber boundary is set to 15% of the reference intakes for a portion of food for ENL and to 25% of the reference intakes for 100 g of food for MTL. In order to avoid additional effects due to non-standardisation of portion sizes, the consortium of companies have proposed standardised portion sizes [22]. Food categories impacted by these modifications are in particular biscuits, chocolate bars, candies, chocolate spreads, i.e., foods generally considered as less healthy. Operationally, this modification leads to more products with amber colours than red colours for less healthy foods.

For the present study, the portion size used to compute the numeric information on the MTL and ENL labels was based on portions sizes officially set by the industry taskforce for the ENL [8]. It allowed to have consistent numeric information in both FoPLs and to investigate only the effect associated with the modification of criteria for colour allocation. For food categories for which a range of portion sizes is proposed in the ENL, the median portion size was used.

More details on the computation of the three FoPLs are provided in Supplementary Material.

#### 2.2.2. Food Categories and Products

The four following criteria were taken into account to select the three food categories for the present study: (i) The food categories had to be commonly consumed in the French population, in order to ensure representative observed behaviours and avoid non-responses due to a lack of familiarity with the products; (ii) food categories had to represent different types of eating occasions—breakfast, snacking and meals; and (iii) food categories were predominantly constituted of products with lower overall nutritional quality (e.g., classified as D or E in the Nutri-Score); and (iv) a standardised portion was recommended by the ENL taskforce. Thus, sweet biscuits, cheeses and sweet spreads were selected. In each of the food category, four different products were chosen to correspond to foods from D or E categories of the Nutri-Score (two products from the D category and two products from the E category, for each food category), with a visible variability in the MTL and ENL labels of the products and in order to be representative of the food offer for the selected food categories.

#### 2.2.3. Portion Sizes

For the three food categories, four photographs of portions sizes were proposed and displayed in increasing order, with a standard difference in grams between portions (i.e., 15 g for spreads and cheese, and one unit of biscuit). Portion sizes selection was based on calibrated photographs, and each photograph corresponded to a standardised portion, depending on the food category. The photographs were calibrated using standard tools (e.g., teaspoon, etc.) and displayed in usual crockery. The size of the crockery displayed was the same, whatever the category of food. Portion sizes proposed to the participants were based on the distribution of the portions consumed for the selected food categories within the NutriNet-Santé study, and on the portion sizes proposed by the ENL Taskforce.

For the category of biscuits, the proposed portion sizes were 1, 2, 3, and 4 biscuits. For cheeses and sweet spreads, consumers were invited to select one of the four photographs corresponding to the following portions: 15 g, 30 g, 45 g and 60 g, containing the recommended portion of ENL (15 g for spreads and 45 g for cheeses).

No additional information was provided, in particular the amount in grams corresponding to the photograph was not provided, to not influence consumers’ choice, in particular as the MTL and ENL provide the portion size within the label.

### 2.3. Procedure

After answering questions on shopping grocery involvement (“Yes”, “No” or “Shared task”), consumption frequency of the tested food categories (“Never”, “Rarely”, “Sometimes”, “Often”, “Everyday”), self-estimated diet quality on a 4-item scale(“I have a very healthy diet”, “I have a mostly healthy diet”, “I have a mostly unhealthy diet” and “I have a very unhealthy diet”), and self-estimated nutrition knowledge level on a 4-item scale (“I am very knowledgeable about nutrition”, “I am somewhat knowledgeable about nutrition”, “I am not very knowledgeable about nutrition” and “I do not know anything about nutrition”), participants were invited to complete the portion size task.

**Figure 2:**
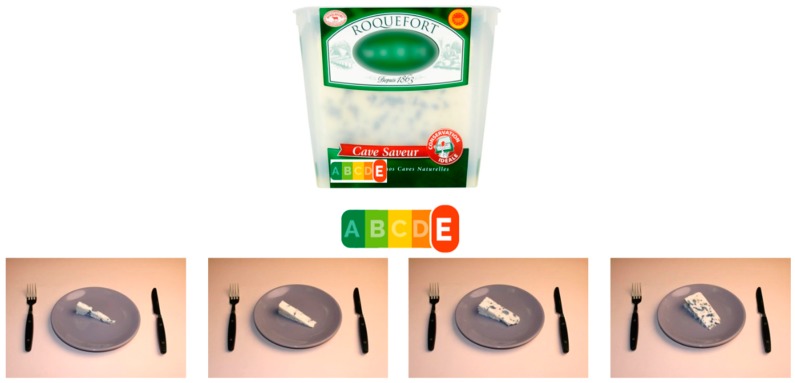
Example of one of the cheese products in the Nutri-Score condition, with the four portion size proposed.

Each participant was exposed to the three food categories and to a set of four products for each category, resulting in the assessment of 12 products overall. They were exposed to the four labelling conditions (ENL, MTL, Nutri-Score, and control without any FoPL) in each set of four products. The order of presentation of the food categories, the order of presentation of the food products and the combination of products*labelling conditions were all randomised, in order to avoid any priming effects. For each food product presented to the subjects, they were invited to select a photograph corresponding to the portion size they would choose to consume, irrespective of their usual consumption. An example of the portion size task is presented in Figure 2. Then, participants had the possibility to select the number of portions (of the size they chose) to indicate the number of portions they would consume on a given eating occasion (between 1 and 4 portions).

### 2.4. Statistical Analyses

All participants who filled and validated the questionnaire were included in the analyses. Sociodemographic and lifestyle characteristics were compared between included and excluded participants, using Student’s *t*-test for continuous variables (age), and Chi square or Fischer test for categorical variable (sex, educational level, occupational activity, monthly income per household unit, marital status, household composition, and smoking status). For each of the 12 products, we considered that the final portion size selected by each participant corresponded to the selected photograph of a portion (using the smallest portion size as one unit) multiplied by the number of portions the participant would consume. Analyses were carried out using the photographs of the portions as units, and not grams, as the quantity corresponding to each photograph was not communicated to participants. Final portion sizes therefore ranged between 1 (e.g., 15 g or 1 biscuit) and 16 (e.g., 240 g or 16 biscuits). Final portion sizes between the four labelling conditions were then compared using Kruskall-Wallis tests (PROC NPAR1WAY in SAS software). Finally, associations between FoPLs and the final portion size were analysed using mixed ordinal logistic regression (PROC GENMOD in SAS software), with the labelling exposure as fixed effect, and the subject as random effect—to take into account intra-individual correlations. The modelled probability was the increase of a portion unit. As randomisation led to a balanced distribution of individual factors across labelling conditions, ordinal logistic regressions were not adjusted. Analyses were performed by food category and then on all food categories combined. Sensitivity analyses were conducted taking into account the consumption frequency of the tested food categories: When subjects answered never consuming a food category, their responses for the corresponding category were excluded. Interaction between FoPLs and individual characteristics, including sex, age, educational level, monthly income per household unit, occupational activity, self-estimated diet quality, nutrition knowledge level, and body mass index (BMI) were tested. Interaction between FoPLs and food categories was also tested.

All tests of significance were two-sided, and a *p*-value of 0.001 was considered significant to take into account multiple testing. Analyses were carried out with SAS software (version 9.4; SAS Institute, Inc., Cary, NC, USA).

## 3. Results

For the present study, 27,198 subjects from the NutriNet-Santé cohort opened the online questionnaire, and 25,772 participants filled and validated their questionnaire, and were then included in the analyses. Individual characteristics of the participants are presented in Table 1. The population study included participants with a mean age of 56.05 ± 14.49 years, 73% of women, 69% of participants with a post-secondary educational level, 23% from managerial staff, 51% with an intermediate level of monthly income (900€–2700€/household unit), 70% in couple, 71% without any children or teenagers living in the household, 12% of smokers, and 34% with a BMI over 25 kg/m^2^. Four percent of included participants estimated having a very unhealthy or unhealthy diet, and 31% being not very knowledgeable or do not know anything about nutrition, and 66% declared being involved in grocery shopping. Individual characteristics between included and excluded participants were globally similar. However included participants tended to be more likely men, with a post-secondary educational level and less from managerial staff, with a higher BMI.

Results of selected final portion size by labelling condition are displayed in Table 2, and association between FoPLs and final portion size are reported in Table 3. Similar results were obtained using a Kruskall-Wallis test, and mixed ordinal logistic regression analyses. A significant interaction between the labels and the food category was observed (*p*-value < 0.0001). For sweet biscuits and cheeses, Nutri-Score followed by MTL significantly led to lower portion sizes compared to no label. The effect of ENL to decrease the portion size was significant for cheeses only (OR = 0.84 (0.83–0.87), *p*-value < 0.0001). However, for sweet spreads results were contrasted according to the FoPL. Indeed, the final portion selected was 1.79 (1.56) for Nutri-Score, followed by MTL (1.91 (1.73)), no label (1.94 (1.70)), and then ENL (2.05 (1.78)). Therefore, compared to no label, Nutri-Score led to the lowest portion size (OR = 0.79 (0.77–0.82), *p*-value < 0.0001), followed by the MTL (OR = 0.94 (0.91–0.97), *p*-value = 0.0001). In contrast, the ENL was associated with a significant increase of the portion size with an OR = 1.19 (1.15–1.22) (*p*-value < 0.0001), compared to no label.

Overall, the mean (SD) final portion size was 1.99 (1.66) portions for the Nutri-Score, followed by MTL (2.05 (1.71)), ENL (2.16 (1.76)) and no label condition (2.17 (1.76)) (overall *p*-value < 0.0001). Compared to no label, the Nutri-Score led to the lowest portion size overall (OR = 0.76 (0.74–0.76), *p*-value < 0.0001), followed by MTL (OR = 0.83 (0.82–0.84), *p*-value < 0.0001), and ENL (OR = 0.99 (0.98–1.00), *p*-value = 0.2).

The mean portions size observed in the study may be partly explained by the fact that approximatively 90% of participants selected a portion size between the four photographs, but kept the number of portions at 1.

In sensitivity analyses, when non-consumers of the food categories were excluded, similar results were observed (Table S1 in the Supplementary Material).

No significant interaction between any individual characteristics and FoPLs was found (all *p*-values > 0.2).

## 4. Discussion

In the present experimental study focusing on three food categories of products for which consumption should be limited, the Nutri-Score was the FoPL which was associated with the lowest portion size selected by consumers, followed by the MTL. However, results for the ENL were inconsistent across food categories. Indeed, this FoPL did not lead to the selection of a lower portion size compared to no label, except for cheeses. Moreover, for sweet spreads the ENL appeared to lead consumers to choose larger portions compared to no label and other FoPLs. In the present study, a portion unit corresponded to 15 g of spreads. Thus, Nutri-Score led to a decrease of 0.15 portions, corresponding to 2.25 g, MTL to a decrease of 0.03 portions, corresponding to 0.45 g and ENL led to an increase of 0.11 portions, corresponding to 1.65 g in any eating occasion compared to no label. Even if observed differences between FoPLs were small in an eating occasion, the high consumption frequency of the tested food categories may lead to a substantial impact of the labels on the portion size consumed over one year for these food products. Therefore, for example for spreads, which are usually consumed at breakfast, assuming one eating occasion per day, compared to no label, Nutri-Score may lead to a decrease of 822 g per year, MTL to a decrease of 164 g per year and ENL to an increase of 603 g per year.

Though information on the recommended portion size for a product (in particular using pictorials) appears to help consumers select an appropriate amount to eat [23,24,25], the scientific literature on the effect of FoPL on portion size perception, such as ours, is very limited. A study investigated the impact of the Health Star Rating system (HSR, a summary FoPL implemented in Australia and New Zealand), compared to an energy only label on portion size in young adults, and reported no significant differences in portion size selection [15]. Another study which compared the effects of the HSR, the Daily Intake Guide label, and MTL on portion size selection, observed a small effect of the HSR to reduce portion size for cornflakes and pizzas only, and an effect of MTL to decrease selected portion sizes for cornflakes only [17], compared to no label. Another study investigated the effect of portion size and caloric Guideline Daily Amounts labelling on portion size choices and consumption of regular soft drinks, but they did not observe any effect on soft drink intake [16]. In the present study, the observed differences of FoPLs effect on portion size perception may be partly explained by their graphical design. Several studies have demonstrated that summary labels providing global information were well perceived and easier to understand compared to nutrient-specific labels. Indeed, the Nutri-Score has been shown to be associated with better perception and higher objective understanding compared to the Multiple Traffic Lights, in general population and specific subgroups [10,11]. According to the findings of this study, the Nutri-Score also seems to help consumers to select more appropriate portions for less healthy foods, by increasing the ability of consumers to identify product healthiness. In contrast, the MTL seemed to perform less well, which may be related to the nutrient-specific design. Nevertheless, it still performed better than the ENL and no label scenario. Interestingly, the Nutri-Score, which does not provide a recommended serving size within the label, as opposed to both MTL and ENL, appeared still to lead to the desired effect of limiting the intake of less healthy foods. Display of portion size is thought to help consumers identifying the appropriate amount to consume, but research suggests that numeric information is less easily accessible that visual cues [26,27]. Our results suggest that the inclusion of a recommended serving size within the label does not appear necessary to lead to a decrease in portion size selection. However, involved mechanisms related to consumers’ perception and behaviour need to be further investigated.

The difference in the performance between the MTL and ENL is likely attributable to the modification of the colour allocation for the ENL (for a portion of product, rather than 100 g, as for the MTL), which appears to lead to somewhat misleading interpretations of the appropriate portion size. Indeed, for some food categories, we observed a limited to no effect for the ENL compared to other labels or no label, to choose the lower portion size of less healthy foods. For spreads, the ENL appeared to be associated to the larger portion size selected by consumers compared to no label and other FoPLs. This could be explained by the colour attribution for the ENL, using less strict threshold compared to MTL and on a per portion basis, which leads to labels without any red for the less healthy products tested in this survey. This is in direct contrast to the Nutri-Score and MTL where scores of “E” and red colour coding were presented to participants for the same products. Thus, with the ENL, consumers are potentially misled about the real nutritional quality of the product and would feel less restraint, resulting in the selection of larger portion sizes, which contradicts the initial objective of the ENL. These results can be considered in the light of studies which found that food labels, which may, sometimes artificially, increase healthiness perception (e.g., “low fat” labelling), could lead to larger portion size selection of less healthy foods [19,28]. However, direct evaluation of healthiness perception was not collected within this study. Further studies investigating specifically the effects associated with the features of the labels (e.g., “D” or “E” for the Nutri-Score, and amber and red colours for the MTL or healthiness perception) are warranted to provide a more detailed assessment of the way consumers respond to labelling in consumption situations.

Furthermore, participants from the present study have selected much larger portions for spreads than the portions considered for the ENL computation (i.e., selecting 2.05 final portions corresponding to 30.79 g on average, compared to the 15 g of the ENL recommended portion), suggesting that such standardised portion sizes are not necessarily realistic compared to current dietary practices. This indicates that presenting nutritional information on a per portion basis is unlikely to contribute to consumers choosing a healthier portion size and may in fact encourage excess consumption in some cases. As such, our results advocate for the presentation of nutritional information per 100 g to help consumers limit overconsumption of less healthy foods. More generally, this study highlights the challenges associated with portion size definition, as they rely on somewhat subjective decisions, with a compromise between defining a ‘recommended’ portion size (i.e., as should be consumed, but may be very different from actual observed behaviour) and an ‘actual’ portion size (i.e., based on consumption studies, but which may illustrate overconsumption for some foods).

Strengths of this study pertained in its large sample size, the selection of different food categories corresponding to various eating occasions, and a variety of food products for each category. In addition, to prevent potential bias of label’s order, a randomised design was used, and to control for a potential bias of food products, all combinations between products and FoPLs were tested and randomised also. Moreover, analyses excluding outliers (i.e., participants who selected a portion size over the 90^th^ percentile of the distribution) were carried out and estimates of FoPLs effects remained similar. However, some limitations should be acknowledged. Firstly, caution is required, regarding the extrapolation of these results to the general French population, since this study included volunteers motivated to participate to nutrition study with probably more health-conscious behaviours and higher socio-professional and educational level overall. Participants of the study were also more likely to have a higher educational level compared to the entire cohort. In addition, repeating the study in the UK or Ireland (where the MTL is well-known among consumers), might produce slightly different results. Secondly, the present study focused on self-reported selection of fictitious portion sizes rather than actual consumption behaviour. Moreover, results might be subject to desirability bias, with subjects selecting purposefully lower portions sizes than their actual consumption. However, participants were blinded to the study outcome, and assessed different foods for each label and the no label condition, which may have limited this bias. Thirdly, some participants, who declared consuming frequently some of the food categories, may have been influenced by their actual usual consumption habits, that could have underestimated the effect of FoPLs. However, given that all participants were exposed to all labelling conditions, this did not impact the difference between FoPLs. Fourthly, the study focused on products with small portions, for which a difference between ENL and MTL would be apparent, but did not compare the FoPLs effects on products with large portion size. Finally, displaying images on an ordinal scale may prompt some subjects to avoid extremes, therefore leading to an over-selection of pictures in the ‘middle range’ (e.g., photographs 2 and 3). Moreover, the limited number of photographs shown to the participants may have increased this bias. However, given that the food products tested in the study are usually consumed in small portions, differences between portions on the four photographs had to be realistic and sufficiently perceivable, limiting us on the number of photographs. Therefore, the results of this study should be interpreted with caution, and be complemented by experimental or in real-life condition studies investigating consumption of real foods and not fictitious products, associated with various FoPLs.

## 5. Conclusions

To conclude, FoP nutrition labels appear to influence portion size selection for less healthy foods, though the magnitude and direction of effects may vary depending on the type of label. The Nutri-Score, and to a lower extent the MTL, appear as efficient tools to decrease the portion size selected by consumers for less healthy products, and therefore help limiting over-consumption of these products. In contrast, the ENL seems to be confusing and misleading on the nutritional quality of products, and may encourage larger portion size selection for some less healthy products, such as sweet spreads. The difference in performance between the MTL and the ENL indicates that the modification/change in boundary for colour coding, which results in fewer reds and more ambers, had a direct impact on portion size perceptions. These findings contradict the hypothesis of the food companies supporting ENL, concerning its potential effect to decrease consumed portion sizes for less healthy products.

## Figures and Tables

**Table 1:** Description of individual characteristics of included and excluded participants.

	Included	Excluded	*p*-Value
***N***	25,772	1426	
**Sex**			0.0005
Men	6966(27.03)	326(22.86)	
Women	18,806(72.97)	1100(77.14)	
**Age, years**	56.05 ± 14.49	56.07 ± 15.13	1.0
**Educational level**			0.05
Primary	4657(18.07)	311(18.18)	
Secondary	3377(13.10)	274(16.01)	
Post-secondary	17,671(68.57)	1096(64.06)	
Missing	67(0.26)	30(1.75)	
**Occupational activity**			0.02
Managerial staff	5806(22.53)	358(25.11)	
Others	19,876(77.12)	1062(74.47)	
Missing	90(0.35)	6(0.42)	
**Monthly income per household unit**			0.3
<900 €	1851(7.18)	103(7.22)	
900 €–2700 €	13,053(50.65)	665(46.63)	
>2700 €	7353(28.53)	409(28.68)	
Missing	3515(13.64)	249(17.46)	
**Marital status**			0.3
In couple	17,989(69.80)	971(68.09)	
Single/divorced/widowed	6785(26.33)	390(27.35)	
Missing	998(3.87)	65(4.56)	
**Household composition**			0.7
Adults only	18,254(70.83)	994(69.71)	
Adults and young children	3792(14.71)	222(15.57)	
Adults and teenagers	1700(6.60)	94(6.59)	
Adults and young children and teenagers	1033(4.01)	51(3.58)	
Missing	993(3.85)	65(4.56)	
**Smoking status**			0.8
Non-smokers	12,490(48.46)	687(48.18)	
Former smokers	9253(35.90)	514(36.04)	
Smokers	3033(11.77)	159(11.15)	
Missing	996(3.86)	66(4.63)	
**Body Mass Index (BMI), kg/m^2^**			0.002
<18.5	1245(4.83)	92(6.45)	
18.5–24	15,667(60.79)	892(62.55)	
25–30	6216(24.12)	320(22.44)	
≥30	2561(9.94)	114(7.99)	
Missing	83(0.32)	8(0.56)	
**Self-estimated diet quality ^a^**			
Very healthy diet	2960(11.49)		
Healthy diet	21,665(84.06)		
Unhealthy diet	1054(4.09)		
Very unhealthy diet	93(0.36)		
**Self-estimated nutrition knowledge level ^a^**			
I am very knowledgeable about nutrition	3585(13.91)		
I am somewhat knowledgeable about nutrition	14,137(54.85)		
I am not very knowledgeable about nutrition	7304(28.34)		
I do not know anything about nutrition	746(2.89)		
**Grocery shopping involvement ^a^**			
No	1898(7.36)		
Yes	17,002(65.97)		
Shared task	6872(26.66)		

^a^ Excluded participants did not answer to these questions.

**Table 2:** Mean portion sizes selected by FoPL (*N* = 25,772).

	Nutri-Score	MTL	ENL	No Label	Pairwise Comparisons between FoPLs ^a^						
					Overall	Nutri-Score vs. MTL	Nutri-Score vs. ENL	Nutri-Score vs. no label	MTL vs. ENL	MTL vs. No Label	ENL vs. No Label
Sweet biscuits	1.99 ± 1.72	2.02 ± 1.71	2.16 ± 1.78	2.18 ± 1.80	<0.0001	0.001	<0.0001	<0.0001	<0.0001	<0.0001	0.4
Cheese	2.19 ± 1.68	2.23 ± 1.69	2.27 ± 1.71	2.39 ± 1.76	<0.0001	0.0003	<0.0001	<0.0001	<0.0001	<0.0001	<0.0001
Sweet spreads	1.79 ± 1.56	1.91 ± 1.73	2.05 ± 1.78	1.94 ± 1.70	<0.0001	<0.0001	<0.0001	<0.0001	<0.0001	0.005	<0.0001
All food categories	1.99 ± 1.66	2.05 ± 1.71	2.16 ± 1.76	2.17 ± 1.77	<0.0001	<0.0001	<0.0001	<0.0001	<0.0001	<0.0001	0.6

^a^*p*-values from pairwise comparisons between FoPLs, using Kruskall-Wallis tests. *p*-value < 0.0001 was considered significant. MTL: Multiple Traffic Lights; ENL: Evolved Nutrition Label.

**Table 3:** Association between portion sizes and FoPLs (*N* = 25,772).

	Sweet Biscuits			Cheese			Sweet Spreads			All Food Categories		
	OR	95% CI	*p*-Value	OR	95% CI	*p*-Value	OR	95% CI	*p*-Value	OR	95% CI	*p*-Value
No label	1			1			1			1		
Nutri-Score	0.73	(0.70;0.74)	<0.0001	0.74	(0.72;0.76)	<0.0001	0.79	(0.77;0.82)	<0.0001	0.76	(0.74;0.76)	<0.0001
MTL	0.77	(0.75;0.79)	<0.0001	0.79	(0.77;0.81)	<0.0001	0.94	(0.91;0.97)	0.0001	0.83	(0.82;0.84)	<0.0001
ENL	0.97	(0.95;1.00)	0.04	0.84	(0.83;0.87)	<0.0001	1.19	(1.15;1.22)	<0.0001	0.99	(0.98;1.00)	0.2

*p*-value < 0.0001 was considered statistically significant. The modelled probability was the increase of a portion unit. OR: Odds Ratio; CI: Confidence Interval; MTL: Multiple Traffic Lights; ENL: Evolved Nutrition Label.

## Supplementary Materials

The following are available online at http://www.mdpi.com/2072-6643/10/9/1268/s1, Table S1: Association between mean portion sizes and FoPLs excluding participants never consuming the tested food categories (*N* = 25,644), Table S2: Attribution of points based on the content of nutrients and other elements per 100g of food, Table S3: Thresholds of FSA score to compute the Nutri-Score, Table S4: Criteria for the colour allocation in MTL and ENL FoPLs.
