# Supplementary material for: Impact of Front-of-Pack Nutrition Labels on Portion Size Selection: An Experimental Study in a French Cohort
Source: Nutrients. 2018 Sep 8;10(9):1268. doi: 10.3390/nu10091268 (PMC6165438; doi:10.3390/nu10091268)
Supplement: Supplementary file 1 [file nutrients-10-01268-s001.pdf]

SUPPLEMENTAL MATERIAL

Table S1. Association between mean portion sizes and FoPLs excluding participants never consuming the tested food categories (N=25,644).

	Sweet biscuits			Cheese			Sweet spreads			All food categories		
	**OR**	**95% CI**	**P-value**	**OR**	**95% CI**	**P-value**	**OR**	**95% CI**	**P-value**	**OR**	**95% CI**	**P-value**
**No label**	1			1			1			1		
**Nutri-Score**	0.72	[0.70;0.74]	<.0001	0.73	[0.71;0.75]	<.0001	0.79	[0.76;0.82]	<.0001	0.75	[0.73;0.76]	<.0001
**MTL**	0.76	[0.75;0.79]	<.0001	0.79	[0.76;0.80]	<.0001	0.95	[0.92;0.98]	0.002	0.83	[0.81;0.84]	<.0001
**ENL**	0.98	[0.95;1.00]	0.07	0.84	[0.83;0.87]	<.0001	1.19	[1.15;1.22]	<.0001	0.99	[0.97;1.00]	0.08

P-value<0.0001 was considered statistically significant. The modelled probability was the increase of a portion unit. .OR: Odds Ratio; CI: Confidence Interval; MTL: Multiple Traffic Lights; ENL: Evolved Nutrition Label The number of consumers having declared never consuming a given food category and who were excluded for sensitivity analyses was 2,200 for sweet biscuits, 945 for cheeses and 3,354 for sweet spreads.

Front-of-Pack Nutrition Labels Computation

Nutri-Score

The Nutri-Score is a summary, graded and color-coded Front-of-Pack nutrition label (FoPL) based on the Food Standard Agency (FSA) Nutrient Profiling System, modified by the Health Council for Public Health. The underlying algorithm of the Nutri-Score allowing the score FSA calculation and the computation of the label is described in the document of the Health Council for Public Health, available at the following link: https://www.hcsp.fr/explore.cgi/avisrapportsdomaine?clefr=519, and summarized below for foods labelling.

Table S2. Attribution of points based on the content of nutrients and other elements per 100g of food

Points	**Points A: For Unfavourable Elements and Nutrients**				**Points C: For Favourable Elements and Nutrients**		
	**Energy (kJ)**	**Saturated Fat (g)**	**Total Sugars (g)**	**Sodium (mg)**	**Fruits, vegetables and nuts[a] (%)**	**Fibre[b] (g)**	**Protein (g)**
0	≤ 335	≤ 1	≤ 4.5	≤ 90	≤ 40	≤ 0.7	≤ 1.6
1	> 335	> 1	> 4.5	> 90	> 40	> 0.7	> 1.6
2	> 670	> 2	> 9	> 180	> 60	> 1.4	> 3.2
3	> 1005	> 3	> 13.5	> 270	-	> 2.1	> 4.8
4	> 1340	> 4	> 18	> 360	-	> 2.8	> 6.4
5	> 1675	> 5	> 22.5	> 450	> 80	> 3.5	> 8.0
6	> 2010	> 6	> 27	> 540			
7	> 2345	> 7	> 31	> 630			
8	> 2680	> 8	> 36	> 720			
9	> 3015	> 9	> 40	> 810			
10	> 3350	> 10	> 45	> 900			

[a] For 100g of a given food, the percentage of fruits and vegetables is obtained by summarising the amount (in grams) of all fruits, legumes and vegetables (including oleaginous fruits, dried fruits and olives) contained in this food.
[b] FSA score allocates different thresholds for fibres, depending on the measurement method used. We used NSP (Non Starch Polysaccharides) cut-offs to compute fibres score.

- If Points A <11, then FSA score = Points A – Points C
- If Points A ≥11,
    - If points for fruits and vegetables = 5, then FSA score = Points A – Points C
    - Else if points for fruits and vegetables <5, then FSA score = Points A – (points for fibre + points for fruits, vegetables and nuts).

Exceptions were made for cheese, fat, and drinks, in order to better rank them according to their nutrient profile, consistently with nutritional recommendations. For cheese, the score takes in account the protein content, whether the A score reaches 11 or not, i.e.: FSA score = Points A – Points C. Fats and drinks are not detailed in the present document.

Then, the FSA score is used to compute the Nutri-Score FoPL with the following thresholds:

Table S3. Thresholds of FSA score to compute the Nutri-Score

Nutri-Score	Foods (100 g)
A	Min - -1
B	0 - 2
C	3 - 10
D	11 – 18
E	19 - Max

Multiple Traffic Lights (MTL)

The Multiple Traffic Lights is a nutrient-specific FoPL based on the original FSA Nutrient Profiling system. A guide describing the computation of this FoPL has been developed by the Department of Health, the Food Standards Agency, and devolved administrations in Scotland, Northern Ireland and Wales, in collaboration with the British Retail Consortium (1).

Evolved Nutrition Label (ENL)

The Evolved Nutrition Label is a nutrient-specific FoPL, based on the MTL graphical format, with a modification for the colour allocation system. The underlying algorithm with the thresholds for colour allocation is described at the following link: https://evolvednutritionlabel.eu/monochrome-to-colours-infographic/.

The details of MTL and ENL computations for foods are summarised below:

Nutrient	Reference Intakes	Low (green)	Medium (amber)	**Multiple Traffic Lights**		**Evolved Nutrition Label**		
				High (red)<br>portion ≤ 100g<br>25% of RIs / 100g	High (red)<br>portion > 100g<br>30% of RIs / portion	High (red)<br>portion ≤ 60g<br>15% of RIs / portion	High (red)<br>60g < portion ≤ 120g<br>25% of RIs / 100g	High (red)<br>portion > 120g<br>30% of RIs / portion
Fat	70g	≤ 3.0g / 100g	> 3.0g to ≤ 17.5g / 100g	> 17.5g / 100g	> 21g per portion	> 10.5g per portion	> 17.5g / 100g	> 21g per portion
Saturated Fatty Acids	20g	≤ 1.5g / 100g	> 1.5g to ≤ 5.0g / 100g	> 5.0g / 100g	> 6g per portion	> 3.0g per portion	> 5.0g / 100g	> 6g per portion
Total sugar	90g	≤ 5.0g / 100g	> 5.0g to ≤ 22.5g / 100g	> 22.5g / 100g	> 27g per portion	> 13.5g per portion	> 22.5g / 100g	> 27g per portion
Salt	6g	≤ 0.3g / 100g	> 0.3g to ≤ 1.5g / 100g	> 1.5g / 100g	> 1.8g per portion	> 0.9g per portion	> 1.5g / 100g	> 1.8g per portion

Table S4. Criteria for the colour allocation in MTL and ENL FoPLs
